# Proteomic Identification of *S*-Nitrosylated Proteins in the Parasite *Entamoeba histolytica* by Resin-Assisted Capture: Insights into the Regulation of the Gal/GalNAc Lectin by Nitric Oxide

**DOI:** 10.1371/journal.pone.0091518

**Published:** 2014-03-13

**Authors:** Rivka Hertz, Shani Ben Lulu, Preeti Shahi, Meirav Trebicz-Geffen, Moran Benhar, Serge Ankri

**Affiliations:** 1 Department of Molecular Microbiology, The Bruce Rappaport Faculty of Medicine, Technion, Haifa, Israel; 2 Department of Biochemistry, The Bruce Rappaport Faculty of Medicine, Technion, Haifa, Israel; University of Hull, United Kingdom

## Abstract

*Entamoeba histolytica* is a gastrointestinal protozoan parasite that causes amebiasis, a disease which has a worldwide distribution with substantial morbidity and mortality. Nitrosative stress, which is generated by innate immune cells, is one of the various environmental challenges that *E. histolytica* encounters during its life cycle. Although the effects of nitric oxide (NO) on the regulation of gene expression in this parasite have been previously investigated, our knowledge on *S*-nitrosylated proteins in *E.histolytica* is lacking. In order to fill this knowledge gap, we performed a large-scale detection of *S*-nitrosylated (SNO) proteins in *E.histolytica* trophozoites that were treated with the NO donor, S-nitrosocysteine by resin-assisted capture (RAC). We found that proteins involved in glycolysis, gluconeogenesis, translation, protein transport, and adherence to target cells such as the heavy subunit of Gal/GalNac lectin are among the *S*-nitrosylated proteins that were enriched by SNO-RAC. We also found that the *S*-nitrosylated cysteine residues in the carbohydrate recognition domain (CRD) of Gal/GalNAc lectin impairs its function and contributes to the inhibition of *E.histolytica* adherence to host cells. Collectively, these results advance our understanding of the mechanism of reduced *E.histolytica* adherence to mammalian cells by NO and emphasize the importance of NO as a regulator of key physiological functions in *E.histolytica*.

## Introduction

Amebiasis is a parasitic infection of the human intestine and is caused by the single-celled protozoa, *Entamoeba histolytica*. The disease has a worldwide distribution with substantial morbidity and mortality, and is one of the three most common causes of death from parasitic disease [1]. The clinical spectrum of amebiasis ranges from asymptomatic infection to colitis, dysentery or liver abscess. The parasite has two stages in its life cycle in the host: the infective cyst and the invasive trophozoite. During its life cycle, the parasite must adapt to various environmental stresses during infection of their human hosts, and the host-parasite relationship is dependent upon the host’s immune system.

Nitric oxide (NO) is a potent cytotoxin, which is released by activated macrophages, natural killer cells, and other phagocytic cells, and has been reported to be the major cytotoxin for killing *E.histolytica* trophozoites [2]. The *S*-nitrosylation of cysteine residues by NO regulates various protein functions, such as enzymatic activity, protein-protein interactions, and protein stability [3], [4]. We have previously reported that NO controls the activity of some of the parasite's virulence factors [2], [5], [6]. It has also been reported that NO triggers stress responses in *E.histolytica* and that NO directly inhibits glycolysis and stimulates cysteine synthase activity [7].

Despite these reports on NO actions on *E.histolytica* trophozoites, our knowledge on *S*-nitrosylated proteins in *E.histolytica* is lacking. Thus, the aim of this investigation is to identify *E.histolytica* proteins that are susceptible to *S*-nitrosylation and to determine their biological relevance in *E.histolytica*. For this purpose, we used resin-assisted capture (RAC) of protein *S*-nitrosothiols (SNO) coupled to mass spectrometry to detect, enrich, and identify *S*-nitrosylated proteins [8]. SNO-RAC was preferred to the biotin-switch assay, a widely-used method for enriching *S*-nitrosylated proteins, because it has fewer steps and was shown to be more efficient for detecting high-mass S-nitrosylated proteins in human embryonic kidney cells that were treated with S-nitrosocysteine (CysNO) [8]. In this analysis of *S*-nitrosylated proteins from *E.histolytica*, we detected 142 *S*-nitrosylated proteins which belong to various metabolic processes, such as catabolism and anabolism, proteolysis, and translation. The heavy subunit of Gal/GalNAc lectin, which is essential for the adherence of the ameba to its target cells [9], was among the *S*-nitrosylated proteins that we identified in NO-exposed *E. histolytica* trophozoites by RAC. We propose that the *S*-nitrosylation of cysteine residues in the carbohydrate recognition domain (CRD) of the heavy subunit of Gal/GalNAc lectin impairs its function and contributes to the inhibition of *E.histolytica* adherence to host cells.

## Materials and Methods

### Microorganisms

*E. histolytica* trophozoites strain HM-1:IMSS were grown under axenic conditions in Diamond's TYI S-33 medium at 37°C. Trophozoites in the exponential phase of growth were used in all experiments.

### Viability Assay under Nitrosative Stress Conditions

*E.histolytica* trophozoites (1×10^6^) were exposed to 100 µM, 500 µM, 1 mM and 2 mM CysNO for 20 minutes. An aliquot (50 µl) of each culture was stained with eosin (0.1% final concentration), and the number of living trophozoites was counted in a counting chamber under a light microscope.

### Detection of Protein *S*-nitrosylation by SNO-RAC

Trophozoites (5×10^7^) that were grown in a Diamond’s TYI-S-33 medium were incubated in freshly-prepared CysNO (500 µM) for 20 minutes at 37°C. A total protein extract was prepared by lysing these CysNO-treated trophozoites with 1% Igepal (Sigma Aldrich) in phosphate buffer saline (PBS). *S*-nitrosylated proteins in the total protein extract were detected by SNO-RAC using a previously described protocol [8] with minor modifications. In brief, the total protein extract (9 mg) was incubated in mixture of 50 mM *N*-ethylmaleimide and 2.5% sodium dodecyl sulfate (SDS) for one hour at 50°C with frequent vortexing in order to block the free thiols. The proteins were then precipitated with three volumes of cold 100% acetone and incubated at −20°C for 20 minutes. The mixture was centrifuged at 1820×g for five minutes, and the pellet was then washed three times with 70% acetone (3 volumes) and then suspended in HENS buffer (HEN buffer which contains 100 mM HEPES, 1 mM EDTA, 0.1 mM neocuproine, and 1% SDS). The suspended samples were added to 80 µl thiopropyl sepharose 6B resin (GE Healthcare) in the presence or absence of sodium ascorbate (final concentration 40 mM). The samples were rotated in the dark for 1–2 hours at room temperature, and then overnight at 4°C. The resin was washed with four times with 1 ml HENS buffer, and then twice with 1 ml HENS/10 buffer (1∶10 HENS buffer). Captured proteins were eluted with 30 µl HENS/10 that contained 100 mM 2-mercaptoethanol for 20 minutes at room temperature, and the proteins in each eluent were resolved on a 12.5% SDS-PAGE gel. Each gel was then stained with Coomassie blue dye (Brilliant Blue G, Sigma Aldrich) and each whole lane was submitted independently for mass spectrometric (MS) analysis.

### In Gel Proteolysis and Mass Spectrometry Analysis

The proteins in each gel slice were reduced with 2.8 mM dithiothreitol (60°C for 30 minutes), modified with 8.8 mM iodoacetamide in 100 mM ammonium bicarbonate (in the dark at room temperature for 30 minutes), and digested overnight in 10% acetonitrile and 10 mM ammonium bicarbonate with modified trypsin (Promega) at 37°C.

The resulting tryptic peptides were resolved by reverse-phase chromatography on 0.075×200-mm fused silica capillaries (J&W) packed with Reprosil reversed phase material (Dr. Maisch GmbH, Germany). The peptides were eluted with linear 95-min gradients of 7–40% and eight minutes with 95% acetonitrile and 0.1% formic acid in water at flow rates of 0.25 µl/min. MS was performed by an ion-trap mass spectrometer (Orbitrap, Thermo) in a positive mode using a repetitively full MS scan followed by collision-induced dissociation (CID) of the seven most dominant ions selected from the first MS scan.

The MS data was analyzed using the Proteome Discoverer software version 1.3 which searches the Ameba section of the NCBI-nr database and the decoy databases (in order to determine the false discovery rate (FDR)) using the **Sequest** and the **Mascot** search engines.

### Classification *S*-nitrosylated *E.histolytica* Proteins according to their Biological Role

The S-nitrosylated proteins were classified according to their biological role using DAVID Bioinformatics Resources (http://david.abcc.ncifcrf.gov/) [10], [11].

### Western Blotting

Total protein extracts were prepared by lysing *E.histolytica* trophozoites (6×10^6^/ml) with 1% Igepal (Sigma) in PBS using a previously described protocol [12]. Proteins (40 µg) in the extract were resolved on a 12% SDS-polyacrylamide gel in SDS-PAGE Running Buffer (25 mM Tris, 192 mM glycine, 0.1% SDS), and then electrotransferred in protein transfer buffer ((25 mM Tris, 192 mM glycine, 20% methanol, pH 8.3) to nitrocellulose membranes (Whatman, Protran BA83). The blots were first blocked using 3% skim milk, and then probed with 1∶500 rabbit polyclonal enolase antibody (Sc-15343; Santa Cruz Biotechnology), 1∶1000 monoclonal glyceraldehyde-3-phosphate dehydrogenase (GAPDH) antibody (MAB374; Millipore), or 1∶500 rabbit polyclonal Gal/GalNAc lectin antibody (a kind gift from of N. Guillen, Pasteur Institute, Paris, France) for 16 hours at 4°C. After incubation with the first antibody, the blots were incubated with 1∶5000 secondary antibody for one hour at room temperature (Jackson ImmunoResearch), and then developed by enhanced chemiluminescence.

### Adhesion Assay

The adhesion of CysNO- or S-nitrosoglutathione (GSNO)-treated trophozoites to HeLa cell monolayers was measured using a previously described protocol [**13**]. Briefly, trophozoites (2×10^5^) were exposed to CysNO (250 µM or 500 µM) or to GSNO (350 µM) for 20 minutes, washed twice with Dulbecco's modified Eagle's medium (DMEM) without serum, added to wells that contained fixed HeLa monolayers in 1 ml of DMEM without serum, and incubated at 37°C for 30 minutes. The number of adherent trophozoites was determined by counting the number of trophozoites that remained attached to the HeLa cells after gentle decanting (twice) of the non-adherent trophozoites with warmed (37°C) DMEM under a light microscope.

### Purification of Gal/GalNAc Lectin by Affinity Chromatography

Trophozoites (1.5×10^7^) were lysed in octyl-beta-d-glucopyranoside (30 mM in water), and D-galactose-coated agarose beads (Thermo Scientific-Pierce Protein Biology Products) were used to bind the Gal/GalNAc lectin in the lysate. After several washes in PBS, the lectin was eluted from the beads using 0.1 M galactose in PBS. The proteins in the eluted fractions were concentrated by ultrafiltration on a Microcon YM-30 column (Millipore) and analyzed by Coomassie blue straining and western blotting using antibody against the heavy subunit of Gal/GalNAc lectin in order to confirm the presence of this lectin in the eluent (data not shown).

### *S*-nitrosylation of Purified Gal/GalNAc Lectin

Aliquots (5 µg) of purified Gal/GalNAc lectin were incubated with cysteine (50 µM) or varying concentration of CysNO (1–500 µM for 20 minutes at 37°C. The Gal/GalNAc lectin was then incubated with 10 µl D-galactose-coated agarose beads (Thermo Scientific-Pierce Protein Biology Products) overnight at 4°C. At the end of the incubation, the beads were washed in 20 volumes of PBS and then boiled in Laemmli sample buffer for 5 minutes. The amount of Gal/GalNAc lectin that was released from the beads was determined using SDS-PAGE gel electrophoresis and silver staining (Thermo Scientific-Pierce Protein Biology Products).

## Results

### SNO-RAC Analysis of *S*-nitrosylated Proteins in *E.histolytica*

**Figure 1:**
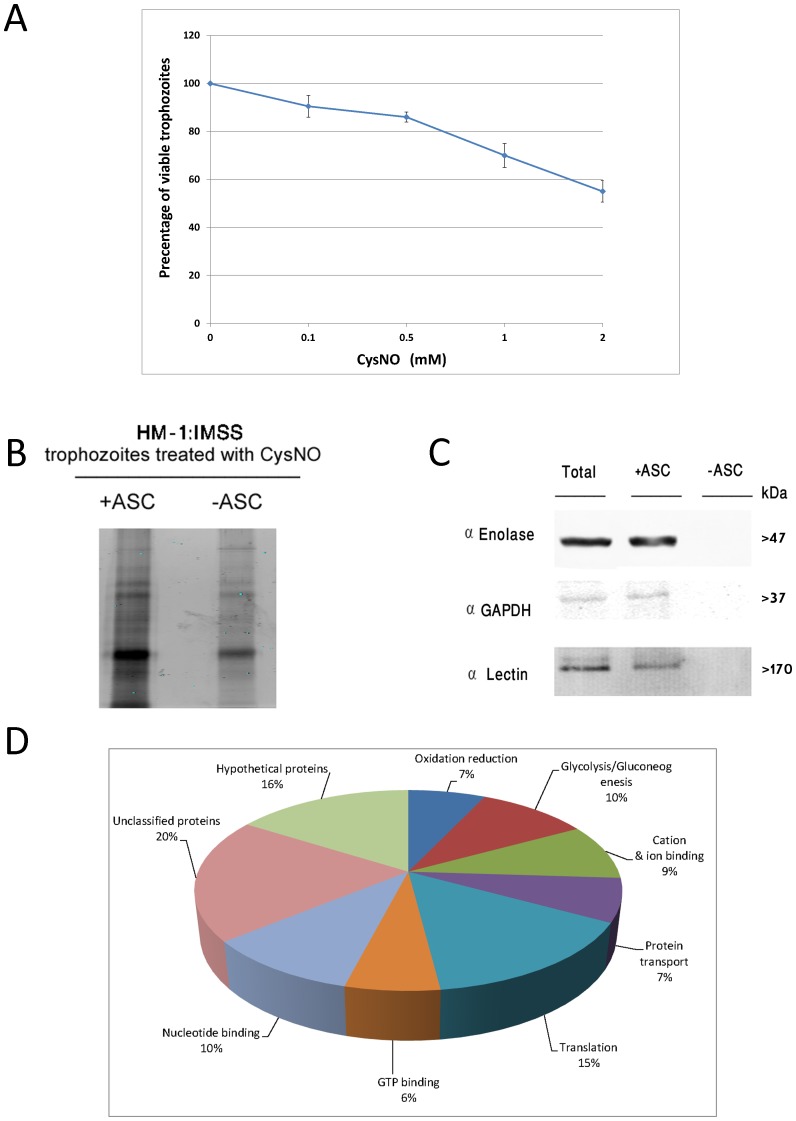
Analysis of *S*-nitrosylated proteins in *E. histolytica* after resin-assisted capture. A. Viability of *E.histolytica* trophozoites which were exposed to different concentrations of S-nitrosocysteine (CysNO) for 20 minutes. Data are expressed as the mean and standard deviation of three independent experiments that were repeated twice. *E.histolytica* trophozoites strain HM-1:IMSS were treated with 500 µM CysNO for 20 minutes. The protein *S*-nitrosothiols (SNO) in the cell lysates was subjected to resin-assisted capture (RAC) in the presence of 40 mM ascorbate (+ASC) or the absence of ascorbate (–ASC). B. Coomassie blue staining of *S*-nitrolysated proteins. C. Functional categories of all *S*-nitrosylated proteins. *S*-nitrosylated proteins in *E.histolytica* were classified according to their biological role. D. Confirmation of *S*-nitrosylation of three proteins, enolase, glyceraldehyde-3-phosphate dehydrogenase, and the heavy subunit of Gal/GalNAc lectin after resin-assisted capture by western blotting. This figure displays a representative result from two independent experiments.

In order to gain information about *S*-nitrosylated proteins in *E.histolytica*, we treated the trophozoites with 500 µM CysNO for 20 minutes. This concentration was chosen because it results in the formation of S-nitrosylated proteins (this work) and only kills 15% of the treated trophozoites (Fig. 1A). Next, we used SNO-RAC coupled to MS to detect, identify, and quantify the *S*-nitrosylated proteins in the lysate of these treated trophozoites. Ascorbate is an SNO reducing agent, which enables the *S*-nitrosylated proteins to bind to the resin by forming disulfide bonds between the reduced thiol groups of the proteins and the thiol group of the resin. We then compared the amounts of *S*-nitrosylated proteins in the untreated and ascorbate-treated (40 mM) lysates (Fig. 1B). A protein was considered to be an *S*-nitrosylated protein when the number of S-nitrosylated proteins in the ascorbate-treated lysates was at least two times greater than that in the untreated lysates. From the results of three independent assays, we found that 142 proteins fulfilled this criterion (see Table S1, Table S2 and Table S3). Those proteins were proteins with diverse functions, such as protein translation, protein transport, and cell metabolism (Fig. 1C). In order to assess the reliability of MS-based identification of the *S*-nitrosylated proteins, three proteins, namely enolase, glyceraldehyde-3-phosphate dehydrogenase (GAPDH), and the heavy subunit of Gal/GalNac lectin, were selected and their *S*-nitrosylation was confirmed by SNO-RAC and western blotting (Fig. 1D). We observed that the amounts of enolase, GAPDH, and the heavy subunit of Gal/GalNac lectin which bound to the thiopropyl sepharose beads were significantly smaller in the untreated samples than the amounts in the ascorbate-treated samples. This result indicates that the binding of those proteins to the thiopropyl sepharose beads did not result from the background affinity of the protein to the beads but depends on its *S*-nitrosylation status (Fig. 1D).

The *S*-nitrosylated proteins were then classified according to their biological function (see Table 1, Table 2, Table 3, Table 4). We found that 10% of the *S*-nitrosylated proteins, such as GAPDH, acetyl-CoA synthetase, alcohol dehydrogenase, enolase, malic enzyme, fructose-1,6-bisphosphate aldolase, and phosphoglycerate kinase, were involved in glycolysis/gluconeogenesis, and various metabolic processes. We also found that 15% of the *S*-nitrosylated proteins were proteins which are involved in translation, such as aspartyl-tRNA synthetase, eukaryotic translation elongation factor 1-alpha (EEF1A1), and various ribosomal proteins. We also found that 6% of the *S*-nitrosylated proteins were proteins that show homology to the superfamily of small GTPases that includes the Ras, Rab, Rho, and Ran subfamilies [14]. We also found that (a) 7% of the *S*-nitrosylated proteins were proteins that involved in free radical detoxification [15], (b) 19% of the *S*-nitrosylated proteins were proteins that involved in ion and nucleotide binding, (c) 20% of the *S*-nitrosylated proteins were proteins that we could not classify, and (d) 16% of the *S*-nitrosylated proteins were hypothetical proteins. Two important *E.histolytica* virulence factors (for a recent review about virulence factors of *E.histolytica* see [16]), the cysteine proteinases (CP) (EhCP2 and EhCP5) and the heavy subunit of the Gal/GalNac lectin, were also present among the *S*-nitrosylated proteins.

**Table 1:** Classification of S-nitrosylated proteins in *Entamoeba histolytica* according to their biological role: Super-family of small GTPase.

GI:	Name	ΣCoverage	MW (kDa)
67470746	Rab family GTPase	32.20	22.9
67482251	Rho GTPase activating protein	5.03	104.9
67469707	Rho family GTPase	26.67	21.6
67481145	Rab family GTPase	40.67	23.4
183231458	rab GDP dissociation inhibitor alpha	11.62	49.3
183235119	GTP-binding protein	12.63	21.5
3420783	Rho GDP exchange inhibitor	13.69	19.3
67477041	Ras family GTPase	17.56	22.7
27923834	RecName: Full = Rho-related protein racG; Flags: Precursor	32.16	21.9

**Table 2:** Classification of S-nitrosylated proteins in *Entamoeba histolytica* according to their biological role: Glycolysis/Gluconeogenesis.

GI:	Name	ΣCoverage	MW (kDa)
67473032	alcohol dehydrogenase 2, putative	54.71	95.5
67481881	acetyl-CoA synthetase	53.58	42.5
67464797	malic enzyme	49.28	53.3
67466006	enolase	43.12	47.3
67479581	alcohol dehydrogenase 3, putative	50.00	42.5
67468656	glyceraldehyde-3-phosphate dehydrogenase	60.48	36.0
67468848	alcohol dehydrogenase, putative	34.99	42.1
67465824	alcohol dehydrogenase putative	37.96	42.5
183232436	malate dehydrogenase	31.23	40.4
183234048	alcohol dehydrogenase, putative	24.11	46.6
67480503	malate dehydrogenase	6.22	40.5
67468719	fructose-1,6-bisphosphate aldolase	30.00	36.2
2317746	PPi-dependent phosphofructokinase	9.71	60.2
67484224	pyruvate phosphate dikinase	38.42	97.8

**Table 3:** Classification of S-nitrosylated proteins in *Entamoeba histolytica* according to their biological role: Translation.

GI:	Name	ΣCoverage	MW (kDa)
67463408	elongation factor 1-alpha 1	57.92	48.4
67467598	aspartyl-tRNA synthetase	36.76	62.6
67471183	asparaginyl-tRNA synthetase	15.30	52.0
67472477	60S ribosomal protein L3	20.40	45.5
67465747	60S ribosomal protein L7	22.61	25.7
67482754	glycyl-tRNA synthetase	8.13	70.6
67465998	leucyl-tRNA synthetase	2.71	122.4
67477437	60S ribosomal protein L6	43.63	22.9
183231631	40S ribosomal protein S4	36.79	35.7
67472614	60S ribosomal protein L10	34.29	23.8
67462433	40S ribosomal protein SA	25.20	28.6
67476939	60S ribosomal protein L10a	10.28	23.8
67478589	40S ribosomal protein S18	15.38	16.3
67465936	60S ribosomal protein L7a	12.94	32.2
67471159	translation initiation factor eIF-5A	12.74	17.1
67465045	60S ribosomal protein L18a	24.28	20.1
67472771	60S ribosomal protein L2/L8	14.79	27.8
67481543	40S ribosomal protein S13	29.05	16.6
67467661	40S ribosomal protein S3	37.30	26.9
399412	Elongation factor 1-alpha	59.53	47.2
57157191	isoleucyl tRNA synthetase	2.42	96.2

**Table 4:** Classification of S-nitrosylated proteins in *Entamoeba histolytica* according to their biological role: Detoxification of free radicals.

GI:	Name	ΣCoverage	MW (kDa)
1888325	pyruvate:ferredoxin oxidoreductase	55.34	127.6
67466737	peroxiredoxin	65.50	25.8
183236094	peroxiredoxin	61.60	26.7
67483345	type A flavoprotein	40.61	46.0
67482769	Fe-hydrogenase	13.46	51.1
67472683	rubrerythrin	17.99	21.2
67465285	Iron-containing superoxide dismutase	22.63	22.0

### Regulation of the Gal/GalNAc Lectin by NO

**Figure 2:**
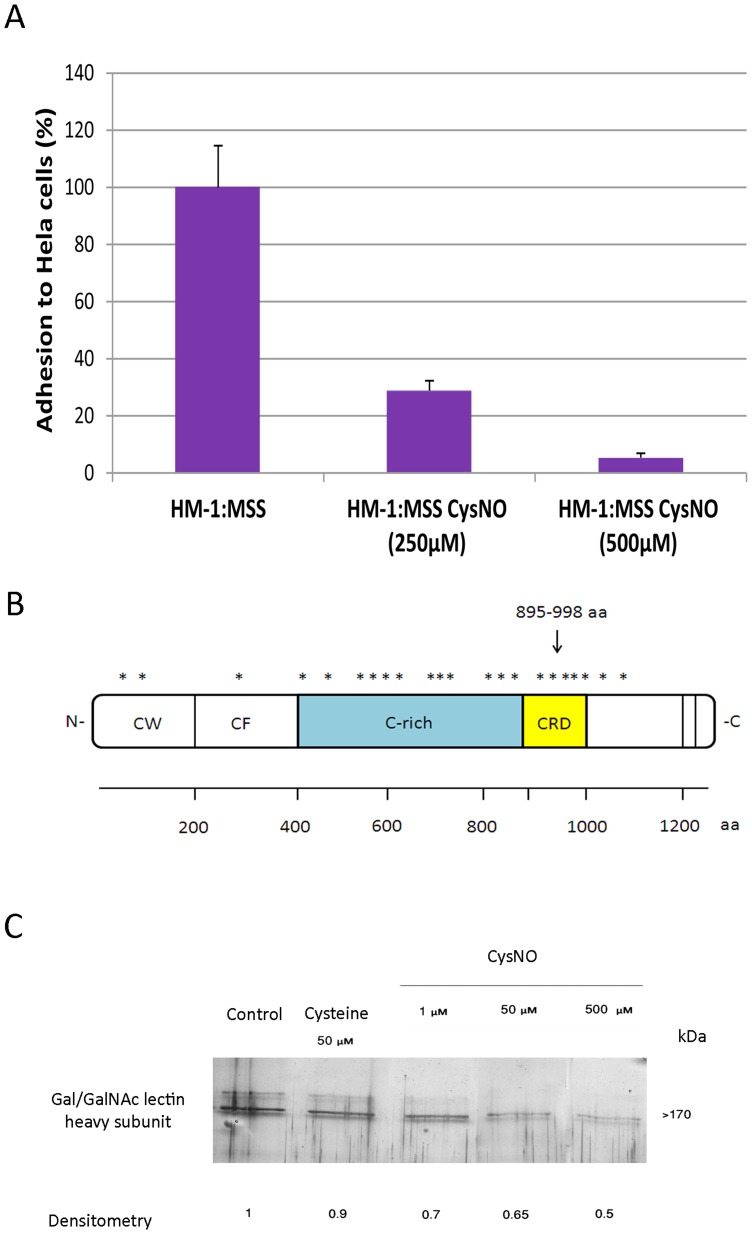
NO inhibits the adhesion of *E. histolytica* to HeLa cells. A. *E.histolytica* trophozoites strain HM-1:IMSS were grown in Diamond's TYI-S-33 medium and exposed to 250 µM or 500 µM S-nitrosocysteine (CysNO) for 20 minutes before their incubation with a fixed HeLa cell monolayer for 30 minutes. The respective value of the control was taken as 100%. Data are expressed as the mean and standard deviation of three independent experiments that were performed in triplicate. The adhesion of the untreated control and CysNO-treated trophozoites was significantly different (p<0.05) according to the results of an unpaired Student's t-test in which statistical significance was set at 5%. B. Repartition of the carbamidomethylated cysteines in the heavy subunit of Gal/GalNac lectin as an indication of their *S*-nitrosylation status. These residues were mostly located in the cysteine-rich region and in the carbohydrate recognition domain. C. Dose-dependent inhibition of Gal/GalNAc lectin binding to D-galactose-coated agarose beads by CysNO. Gal/GalNAc lectin was purified by D-galactose affinity chromatography and incubated with different concentrations of CysNO for 20 minutes. The binding to galactose-coated agarose beads was determined by SDS-PAGE gel electrophoresis and silver staining. For the negative control, Gal/GalNAc lectin was incubated with 50 µM cysteine prior to its interaction with the galactose beads. This figure displays a representative result from two independent experiments.

Gal/GalNac lectin is a 260-kDa heterodimer and a cell surface molecule which is involved in the recognition and adhesion of *E. histolytica* to a distinct receptor on host cells [17], [18]. The molecular mass of the disulfide-linked heavy subunit of this lectin is 170 kDa and the molecular mass of the light subunit of the lectin is 35/31 kDa. The heavy subunit mediates *E. histolytica* adherence, and there is indirect evidence that the light unit is involved in *E. histolytica* virulence ([13], [19]. The presence of the heavy subunit of the Gal/GalNac lectin among the S-nitrosylated proteins (see Table S2) suggests that NO regulates the adherence of the parasite to mammalian cells. In order to test this hypothesis, we compared the ability of untreated and CysNO-treated trophozoites (2×10^5^) to bind to a fixed HeLa cell monolayer [13]. We also compared the viabilities of the untreated and CysNO-treated trophozoites after incubating the trophozoites to either 250 µM or 500 µM CysNO for 20 minutes before their 30-minute incubation with the HeLa cell monolayer. We found that the binding of the CysNO-treated trophozoites to the HeLa cell monolayer was significantly less (70% and 95%, respectively) than that of the untreated control trophozoites (Fig. 2A). We also found that the viability of the CysNO-treated trophozoites was 15% lower than that of the untreated control trophozoites (data not shown). From these results, we concluded that the reduced adherence of the parasite to the HeLa cell monolayer cannot be attributed to a lethal action of CysNO. In order to confirm this conclusion, we repeated this assay using another NO donor drug, GSNO (350 µM), and found that GSNO also reduces the adherence of the parasite to the HeLa cell monolayer and it has a mild effect on the viability of the GSNO-treated trophozoites: the viability was 15% lower than that on the untreated control trophozoites) (data not shown).

Results from previous investigations indicate that the cysteine-rich region of the heavy subunit of Gal/GalNAc lectin and more specifically the CRD are essential for the binding activity of the lectin [19], [20]. The results of the MS analysis of the heavy subunit of Gal/GalNAc lectin that was bound to the thiopropyl sepharose resin in the presence of sodium ascorbate showed that numerous carbamidomethylated cysteine residues are located in the cysteine-rich region and the CRD of the lectin (Fig. 2B). These residues possibly correspond to *S*-nitrosylated cysteines that have been reduced by the ascorbate, bound to the resin, eluted by 2-mercaptoethanol, and alkylated by iodoacetamide prior to digestion of the protein and MS analysis.

In order to demonstrate that *S*-nitrosylation of the Gal/GalNAc lectin inhibits its carbohydrate binding ability, purified Gal/GalNAc lectin was incubated 1 µM, 50 µM and 500 µM of CysNO for 20 minutes and their binding ability to galactose-coated agarose beads was determined by SDS-PAGE gel electrophoresis and silver staining. We found that CysNO inhibits the binding of the Gal/GalNAc lectin to the galactose beads in a dose-dependent manner (Fig. 2C). In contrast, no inhibition was observed when the Gal/GalNAc lectin was incubated with cysteine prior to their exposure to the galactose-coated agarose beads.

## Discussion

NO is one of the most versatile molecules in the immune system [21] and is released during infections by unicellular parasites, viruses, and bacteria. One of the mechanisms of NO-mediated cytotoxicity relies upon the formation of *S*-nitrosothiols, which then react with sulfhydryl groups in proteins and results in abnormal protein function and cell stasis or death. Previous reports in *E.histolytica* have shown that NO inhibition of glycolytic enzymes in *E.histolytica* leads to fragmentation of the endoplasmic reticulum, reduction of ATP levels, and parasite death [7]. NO also inhibits CPs [6], which are involved in differentiation, amino acid anabolism, inactivation of the host inflammatory response, lysosomal transport, and invasion of the host’s tissues [22], [23], [24], [25], [26], [27]. Although the results of these abovementioned studies have greatly contributed to our understanding of the antiamebic effect of NO, these studies were restricted in that they only investigated NO actions on a limited number of the *E.histolytica*’s proteins. Accordingly, we decided fill this knowledge gap by performing a high-throughput proteomic analysis of *S*-nitrosylated proteins in the parasite after its exposure to NO. Since SNO-RAC is a method which is able to confirm the identity of previously reported *S*-nitrosylated proteins [6], [7], we used SNO-RAC for investigating the existence of *S*-nitrosylated proteins in *E.histolytica*. Although we cannot exclude that some of the SNO proteins may have been lost during the SNO-RAC procedure, we were able to identify many novel *S*-nitrosylated proteins in the parasite.

The first group of *S*-nitrosylated proteins that we identified in our SNO-RAC analysis is a group of 14 proteins that are involved in energy metabolism. Numerous glycolytic enzymes were also found to be S-nitrosylated in *Plasmodium falciparum* [28]. In both *P.falciparum* [28] and *E.histolytica* [7], their S-nitrosylation usually leads to the inhibition of their enzymatic activity and consequently to a reduction of energy metabolism. Remarkably, we found that one-third of these *E.histolytica*'s S-nitrosylated proteins are proteins which are linked to energy metabolism and belong to the family of alcohol dehydrogenases (ADH), namely ADH2 (GI 67473032), ADH3 (GI 67479581), and an unclassified ADH (GI 183234048). ADH2 is an essential enzyme for the growth and survival of the parasite [29], [30] and its inhibition by NO [6] probably contributes to NO's antiamebic activity.

In this group, we found GAPDH is another *S*-nitrosylated protein. In mammals, this protein has moonlight activity and in addition to its glycolytic function, it can initiate apoptosis following its *S*-nitrosylation [31]. This protein has also be involved in the defense against both reactive oxygen species and nitrogen intermediates in *Escherichia coli* [32] and its overexpression in *Leishmania infantum* amastigotes confers NO resistance [33]. In contrast to *P. falciparum* or mammalian GAPDH [28], [34], GAPDH activity in *E.histolytica* is not modified by NO [7] after its S-nitrosylation. In fact, the results of our SNO-RAC screen suggest that this enzyme is an NO scavenger in *E.histolytica*. The presence of GAPDH on the parasite's surface [35] supports the notion that this enzyme is an NO scavenger in *E.histolytica* and confirmatory studies are now necessary.

The second group of *S*-nitrosylated proteins that we identified in our SNO-RAC analysis is a group of proteins which are involved in protein translation, such as EEF1A1 and various ribosomal proteins. It is well known that NO inhibits total protein synthesis and mammalian cell proliferation [36]. We also found that total protein synthesis was inhibited in GSNO- or CysNO-treated trophozoites (data not shown; manuscript in preparation). EEF1A1 is responsible for the delivery of aminoacyl transfer RNAs to the ribosome. Since this protein is essential for protein synthesis in mammals, its inhibition results in cell death [37]. This protein has also been found to be *S*-nitrosylated in prostate epithelial cells after their exposed to NO [38]. Moreover, it has been proposed that *S*-nitrosylation of the 40S and 60S subunit ribosomal proteins impairs protein synthesis in plants [39]. Collectively, the reduced protein synthesis that we found in the NO-treated parasites can be attributed to *S*-nitrosylation of elongation factor 1-alpha 1 and some ribosomal proteins.

The third group of *S*-nitrosylated proteins that we identified in our SNO-RAC analysis is a group of protein homologs to the superfamily of small GTPases [14]. These proteins are signaling molecules that are activated by various extracellular stimuli [14]. In *E. histolytica*, a unique Rab GTPase, EhRabA, is involved in the parasite’s motility and polarization [40] and in the localization of Gal/GalNAc lectin [41]. Saito-Nakano and colleagues [42] reported that two Rab7 isotopes have distinct roles in the biogenesis of lysosomes and phagosomes in *E. histolytica*. It has also been reported that some Rab GTPase genes differentially respond to H_2_O_2_ and NO, and such responses suggest a possible link between the response to oxidative or nitrosative stress and membrane trafficking [43], [44]. Although the exact nature of this link is still not understood, these results suggest that *S*-nitrosylation of Rab proteins regulates their function.

The fourth group of *S*-nitrosylated proteins that we identified in our SNO-RAC analysis is a group of proteins that are involved in detoxification of free radicals [15]. These *S*-nitrosylated proteins are peroxiredoxin, ruberythrin, superoxide dismutase, and type A flavoprotein. These proteins are involved in the detoxification of H_2_O_2_ and the superoxide radical and in the reduction of oxygen and NO that is released in nitrosative stress. It has been recently reported that NO upregulates the expression of the gene that encodes for peroxiredoxin and three genes that encode for iron-sulfur-flavoprotein [7]. Collectively, these results suggest that NO regulates crucial components of the detoxification pathways of reactive oxygen species and reactive nitrogen species. Accordingly, we posit that the functions of these S-nitrosylated proteins in the NO-exposed *E.histolytica* trophozoites are important for the parasite’s survival under conditions of oxidative and/or nitrosative stress.

Another group of *S*-nitrosylated proteins that we identified in our SNO-RAC analysis is a group of CPs. These CPs are essential virulence factors and they have been intensively studied in *E.histolytica* [26], [27], [45]. Two CPs, EhCP-2 and EhCP-5, were among the proteins enriched by SNO-RAC. Their expression levels have been reported to be correlated with the level of destruction of a mammalian cell monolayer by the parasite (cytopathic activity) and its ability to form liver abscesses in hamsters [27], [46], [47]. We have previously reported that *S*-nitrosylation of *E.histolytica* CPs inhibits their activity [6] and impairs the parasite’s virulence. *P.falciparum*’s virulence is also impaired by the *S*-nitrosylation of falcipain proteinases [28] [48], which are also CPs, and falcipain proteinases are involved in the hydrolysis of host erythrocyte hemoglobin in the parasite food vacuole [49].

The Gal/GalNAc lectin is a central virulence factor of *E.histolytica* which mediates the adherence of the parasite to its target cells. The heavy subunit of Gal/GalNAc lectin includes a cysteine-rich (C-rich) region (amino acids 356–1143) and a CRD (amino acids 895–998) that are essential for its binding activity [20]. Based on our experimental results, we proposed that the *S*-nitrosylation of cysteine residues in the CRD is sufficient to inhibit the galactose binding activity of the Gal/GalNAc lectin and contributes to the reduced binding of NO-treated trophozoites to their target cells. Interestingly, the C-rich region of the Gal/GalNAc lectin triggers an inflammatory process that results in the production of NO by gamma-primed bone marrow macrophages [50], [51]. Further experiments are needed to determine whether *S*-nitrosylated Gal/GalNAc lectin is still able to induce NO production or whether its S-nitrosylation is part of a regulatory process to downregulate inflammation following the neutralization of the parasite. Other proteins, such as the serine-rich *E. histolytica* protein [52] and the *E. histolytica* EhCPADH complex [53], which comprises a CP and an adhesin, are also involved in the adherence of the parasite to mammalian cells. Although these proteins were not identified in our SNO-RAC analysis, it is possible that they have been missed by this enrichment procedure and that their *S*-nitrosylation may contribute to the reduced adherence of the parasite to mammalian cells.

To conclude, this investigation informs on the results of the first global analysis of *S*-nitrosylated proteins in *E.histolytica*, and thus, paves the way for further studies on the effect of *S*-nitrosylation on the activity of the identified proteins. From the results of this investigation, we have also discovered an unexpected action of NO in *E.histolytica*, namely NO inhibits Gal/GalNAc lectin activity and its adherence to mammalian cells.

## Supporting Information

Table S1 List of all *S*-nitrosylated proteins that were enriched by resin-assisted capture in three independent experiments.
(XLSX)

Table S2 List of S- nitrosylated proteins that were enriched at least two times or more by resin-assisted capture in three independent experiments.
(XLSX)

Table S3 Description of the parameters that are given in Table S1 and Table S2.
(DOCX)
