# Supplementary material for: Proteomic Identification of S-Nitrosylated Proteins in the Parasite Entamoeba histolytica by Resin-Assisted Capture: Insights into the Regulation of the Gal/GalNAc Lectin by Nitric Oxide
Source: PLoS One. 2014 Mar 13;9(3):e91518. doi: 10.1371/journal.pone.0091518 (PMC3953491; doi:10.1371/journal.pone.0091518)
Supplement: Table S3 — Description of the parameters that are given in Table S1 and Table S2. (DOCX) [file pone.0091518.s003.docx]

| Feature | Description |
| --- | --- |
| Accession | Displays by default the unique identifier assigned to the protein by the FASTA database used to generate the report. |
| Description | Provides the name of the protein exclusive of the identifier that appears in the Accession column. This description appears in the table by default. |
| Coverage | Displays by default the percentage of the protein sequence covered by identified peptides. |
| # Proteins | Displays the number of identified proteins in the protein group of a master protein. This number is the same as that displayed in the Protein Group Members view when you choose Search Report > Show Protein Group Members View (see [this figure](mk:@MSITStore:C:\PROGRA~1\Thermo\DISCOV~1.3\System\Release\THERMO~1.CHM::/Interpreting%20Your%20Results%20with%20the%20Protein%20Group%20Members%20View.htm#1356604)). |
| # Unique Peptides | Displays the number of peptide sequences unique to a protein group. |
| # Peptides | Displays the number of distinct peptide sequences in the protein group. |
| # PSMs | Displays the total number of identified peptide sequences (peptide spectrum matches) for the protein, including those redundantly identified. |
| # AAs | Shows by default the sequence length of the protein. |
| Score | Displays the protein score, which is the sum of the scores of the individual peptides.   \| • \| For SEQUEST results, the score is the sum of all peptide Xcorr values above the specified score threshold. The score threshold is calculated as follows: \| \| --- \| --- \|   0.8 + peptide_charge × peptide_relevance_factor  where peptide_relevance_factor is an advanced parameter of the SEQUEST node in the “Protein Scoring Option” category with a default value of 0.4. For each spectrum, only the highest-scoring match is used.  For each spectrum and sequence, the Proteome Discoverer application uses only the highest scored peptide. When it performs a search using dynamic modifications, one spectrum might have multiple matches because of permutations of the modification site.   \| • \| For ZCore results, the score is the highest probability score of all peptides identified for that protein. \| \| --- \| --- \| |
| Score, continued | \| • \| For Mascot results, the score is: \| \| --- \| --- \| \| – \| Standard score, which is the cumulative protein score based on summing the ion scores of the unique peptides identified for that protein. If a peptide was redundantly identified, only the highest-scoring peptide is used. \|   –or–   \| – \| MudPIT score, which is the sum of the “excess of ions” score over the homology or identity threshold for each spectrum plus the average threshold. For MudPIT scoring, the score for each peptide is not its absolute score but the amount that it is above the threshold. Therefore, peptides with a score below the threshold do not contribute to the score. For each peptide, the threshold is the homology threshold, if it exists; otherwise, it is the identity threshold. \| \| --- \| --- \|   By default, the Proteome Discoverer application automatically switches between the standard and the MudPIT score to calculate the protein score in the Mascot node results. It automatically uses the MudPIT score when the number of queries divided by the number of FASTA database entries exceeds 0.001. |
| MW [kDa] | Displays the calculated molecular weight of the protein. The Proteome Discoverer application calculates the molecular weight without considering PTMs.  Separating proteins by molecular weight can be one of the steps in two-dimensional gel electrophoresis. You can use the protein’s molecular weight as a rough constraint to estimate whether it is reasonable to identify a particular protein in a certain fraction that was searched. |
| calc. pI | Displays the theoretically calculated isoelectric point, which is the pH at which a particular molecule carries no net electrical charge.  The amino acids that make up proteins can be positive, negative, neutral, or polar in nature, and together they give a protein its overall charge. At a pH below their isoelectric point, proteins carry a net positive charge; at a pH above their isoelectric point, they carry a net negative charge. Gel electrophoresis can then separate proteins according to their isoelectric point (overall charge) with a polyacrylamide gel, using a technique called isoelectric focusing, which uses a pH gradient to separate proteins. Isoelectric focusing is also the first step in two-dimensional gel polyacrylamide gel electrophoresis.  When you have searched the fractions resulting from isoelectric focusing, you can use the calc. pI value to estimate whether you might expect to find a particular protein in the given fraction. |
| Area (in MSF files with isotopically labeled quantification results only) | Displays the average area of the three unique peptides with the largest peak area. |
